# Frailty influences clinical outcomes in critical patients: a post hoc analysis of the PalMuSIC study

**DOI:** 10.62675/2965-2774.20250229

**Published:** 2025-05-07

**Authors:** Ana Mestre, Rodrigo Afonso, André Ferreira-Simões, Iuri Correia, João Gonçalves Pereira

**Affiliations:** 1 Hospital Vila Franca de Xira Intensive Care Unit Vila Franca de Xira Portugal Intensive Care Unit, Hospital Vila Franca de Xira - Vila Franca de Xira, Portugal.; 2 Universidade de Lisboa Faculdade de Medicina Lisboa Portugal Faculdade de Medicina, Universidade de Lisboa - Lisboa, Portugal.; 3 Hospital CUF Tejo Palliative Care Unit Lisboa Portugal Palliative Care Unit, Hospital CUF Tejo - Lisboa, Portugal.

**Keywords:** Frailty, Frail elderly, Clinical frailty scale, Mortality, Critical care outcomes, Length of stay, Intensive care units

## Abstract

**Objective:**

Frailty is a multidimensional syndrome characterized by diminished physiological reserve, increasing the risk of adverse outcomes, particularly in intensive care unit patients. The Clinical Frailty Scale, ranging from 1 (nonfrail) to 9 (terminally ill), is widely used to quantify frailty. This post hoc analysis of the Palliative Multicenter Study in Intensive Care (PalMuSIC) assesses the impact of frailty and clinical severity on short- and long-term outcomes.

**Methods:**

This subanalysis involved 23 Portuguese intensive care units and 335 patients. Patients admitted between March 1 and May 15, 2019, aged ≥ 18 years, and hospitalized for > 24 hours in the intensive care unit were eligible. The severity of illness was assessed using SAPS II, and frailty was assessed using the clinical frailty scale, which was recorded by a nurse and a doctor in charge. Patients were classified as frail (clinical frailty scale score ≥ 5), prefrail (clinical frailty scale score = 4), or nonfrail (clinical frailty scale score < 4). The outcomes measured included intensive care unit and hospital LOS (length of stay), need for organ support, infections, mortality at hospital discharge and mortality at 6 months post discharge. We divided the population in half according to the length of their intensive care unit stay to evaluate a possible interaction between intensive care unit length of stay and frailty.

**Results:**

The mean age was 63.2 years, and 66% were male. The mean SAPS II score was 41.8. Frailty was observed in 23.0% of the patients. Frail patients had higher hospital mortality (39.0% frail patients *versus* 28.2% prefrail patients *versus* 11.8% nonfrail patients) and 6-month mortality (frail 49.4% frail patients *versus* 30.6% prefrail patients *versus* 15.6% nonfrail patients). Patients with longer intensive care unit stays had higher 6-month mortality rates than did those with shorter intensive care unit stays did, which resulted in more frail patients: odds ratio (95% confidence interval) 3.1 (1.2 - 7.8) *versus* odds ratio 1.8 (0.9 - 4.0) in nonfrail patients.

**Conclusion:**

Frailty may significantly impact hospital and 6-month mortality. In our cohort, a longer intensive care unit length of stay was associated with worse long-term outcomes, especially in frail patients.

## INTRODUCTION

Frailty is a complex and multidimensional syndrome characterized by diminished physiological reserve, increasing the risk of poor outcomes for patients.^(1)^ It is a condition of vulnerability that reduces the ability to adapt to an external insult or recover after acute illness. The size of the senior population is increasing worldwide, making frailty an increasing concern for healthcare systems. Although frailty is more prevalent in elderly individuals, it is not invariably associated with age and exists among all age groups. Frailty is common in patients admitted to the intensive care unit (ICU), and it is associated with worse outcomes.^(2)^

The Clinical Frailty Scale (CFS) is widely used in the ICU population to quantify frailty. It is a clinical judgment-based assessment tool consisting of a rating scale designed to assess frailty according to physical activity, functional status, chronic illness burden, and cognition. This score uses a scale that ranges from 1 to 9. Scores of 1 to 3 are considered nonfrail; a score of 4 is considered prefrail or vulnerable; scores of 5 to 8 are deemed frail; and a score of 9 indicates terminal illness.^(3)^ The prevalence of frailty in critically ill patients may be as high as 35%,^(4)^ dependent on the type of ICU. Frailty assessed by the CFS is strongly correlated with adverse outcomes.^(3,5)^ Preadmission frailty has been identified as a relevant risk factor in critically ill patients for ICU- and hospital-related mortality, as well as for prolonged ICU hospitalization.^(4,6)^

Clinical severity scores are largely available in the ICU setting and allow the evaluation of different populations’ severity and death probability as surrogates of the quality of care for benchmarketing purposes. Scoring systems, such as the commonly used Simplified Acute Physiology Score (SAPS) II, help to describe ICU populations and interpret outcome measures. The interactions among frailty, severity scores and outcomes are poorly understood.

This study is a post hoc analysis of a multicenter, prospective, observational study, the PALliative MUlticenter Study in Intensive Care (PalMuSIC).^(7)^ We aimed to assess the impact of the interaction between frailty and clinical severity on short- and long-term outcomes. We also wanted to address whether a prolonged ICU length of stay (LOS) may worsen the outcomes of the frail population due to their lower physiological reserve.

## METHODS

This is a subanalysis of the PalMuSIC study. The study protocol has been published elsewhere.^(7)^ Briefly, PalMuSIC was a prospective, observational, multicenter study carried out to evaluate the prevalence of frailty in Portuguese ICUs and the use and adequacy of palliative care and invasive interventions.

The study was conducted in 23 Portuguese ICUs and included 335 patients. All patients admitted during a 15-day consecutive period, starting between 1st March and May 2019, aged ≥ 18 years and hospitalized for > 24 hours in the ICU, were considered eligible for inclusion. Written informed consent was obtained from all patients or their representatives.

The severity of illness at admission was assessed according to SAPS II, and frailty was assessed using the CFS. The CFS was independently recorded by the nurse and the doctor in charge. According to the average of these two assessments, all patients were classified as frail (CFS ≥ 5), prefrail (CFS = 4), or nonfrail (CFS < 4). We also asked the family member with the closest relationship to the patient to provide an assessment of the CFS. The ICU and hospital LOSs were calculated, along with the need for organ support (noninvasive or invasive mechanical ventilation, renal replacement therapy, or vasopressors). Infection upon admission or acquired in the ICU was also recorded. Mortality was evaluated at discharge from the hospital and at 6 months after discharge.

### Outcomes

We developed a multivariable model to identify risk factors independently associated with hospital mortality and 6-month all-cause mortality. To assess the interaction between a prolonged ICU LOS and frailty, we further divided our population into short and long ICU LOSs according to the median ICU LOS (5 days).

### Statistics

The data are summarized as the means ± standard deviations or medians [25 - 75% interquartile ranges (IQRs)] according to the data distribution. Categorical variables are described as N (%). The chi-square test was used to compare categorical variables, while continuous variables were evaluated with Student's *t* test or the Kruskal—Wallis test, according to the data distribution. Multiple comparisons of variance were assessed with ANOVAs. Odds ratios (ORs) with 95% confidence intervals (95%CIs) were computed. We developed a multivariable model that included comorbidities, the SAPS II score, frailty status and ICU LOS to assess factors independently associated with hospital mortality and 6-month all-cause mortality. The univariate associations of clinically significant variables were assessed for model development. To ensure the inclusion of all clinically significant variables in the model, a p value as high as 0.2 was used for selection. Moreover, if an excluded variable was considered to have a possible influence on the outcome, it was also included in the model. Correlations between all included variables were checked. We arbitrarily used r < 0.3 as a low enough threshold to decrease the risk of significant multicollinearity. For variables that were correlated, the one that was considered more likely to be related to the outcome was selected. Model fit was assessed with the Hosmer—Lemeshow goodness-of-fit test. Statistical analysis was performed using IBM SPSS Statistics v.29.0 (IBM, Somers, NY, USA). All statistics were 2-tailed, and the significance level was p < 0.05.

## RESULTS

### Demographics

A total of 335 patients were included. The mean age was 63.2 ± 16.8 years, and 66% were male. The mean SAPS II value was 41.8 ± 17.4. The general characteristics of the patients are shown in table 1.

**Table 1:** Demographic and general characteristics of the population

	Total	Frail	Prefrail	Nonfrail	p value*
Patients	335	77 (23.0)	85 (25.4)	173 (51.6)	
Male	221 (66.0)	52 (67.5)	49 (57.6)	120 (69.4)	0.166
Age	63.2 ± 16.8	71.5 ± 12.8	68.4 ± 13.1	56.9 ± 17.5	< 0.001†
ICU LOS	5 [7]	5 [8]	5 [6.5]	6 [9.5]	0.765‡
SAPS II	41.8 ± 17.4	50.0 ± 17.4	45.7 ± 16.0	36.3 ± 16.1	< 0.001†
Infection	201 (60)	46 (59.7)	48 (56.5)	107 (61.8)	0.731
IMV	214 (63.9)	50 (64.9)	59 (69.4)	105 (60.7)	0.382
NIV	57 (17.0)	15 (19.5)	20 (23.5)	22 (12.7)	0.076
RRT	48 (14.3)	17 (22.1)	15 (17.6)	16 (9.2)	0.017
Vasopressors	185 (55.2)	48 (62.3)	50 (58.8)	87 (50.3)	0.155
Previous hospital admission (3 months)	75 (22.4)	27 (35.1)	18 (21.2)	30 (17.3)	0.08
Hospital readmission (6 months)§	74 (28.9)	22 (46.8)	22 (36.1)	30 (20.3)	< 0.001

ICU - intensive care unit; LOS - length of stay; SAPS - Simplified Acute Physiology Score; IMV - invasive mechanical ventilation; NIV - noninvasive ventilation RRT - renal replacement therapy.

* Chi-square test except

† ANOVA with Fisher's variance method and

‡ Kruskal-Wallis test;

§ Patients discharged alive from the hospital only. Data are presented as the n (%), mean ± standard deviation or median [interquartile range] according to the data distribution.

Frailty was observed in 23.0% of patients. Frail, prefrail, and nonfrail patients were significantly different in terms of age and SAPS II score (Table 1).

### Mortality and standardized mortality ratio

Frail patients had higher in-hospital mortality rates (39.0% *versus* 28.2% *versus* 11.8%; p < 0.001). All-cause mortality after 6 months of follow-up was also significantly greater in frail patients (49.4% *versus* 30.6% *versus* 15.6%, p < 0.001) (Table 2).

**Table 2:** Hospital and 6-month mortality

	N	Hospital mortality*	6-month mortality*
Nonfrail	173	24 (11.8)	34 (15.6)
Prefrail	85	24 (28.2)	26 (30.6)
Frail	77	30 (39.0)	38 (49.4)

Data are presented as n (%).

* Chi-square test, p < 0.001.

In addition, frail patients who survived until hospital discharge often needed hospital readmission in the first 6 months, and this number was significantly higher than that of nonfrail patients (Table 1).

**Figure 1:**
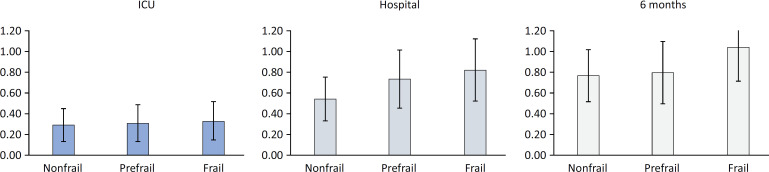
Standardized mortality ratios, with 95% confidence intervals, calculated at intensive care unit discharge, hospital discharge, and after 6 months of follow-up, revealing the potential influence of frailty on mortality.

In figure 1, we present the standardized mortality ratio (with 95%CI), which was calculated as the ratio between the observed mortality and the SAPS II score-predicted mortality on admission to the ICU. Frailty seemed to impact the standardized mortality ratio, revealing the importance of the case mix on the outcomes.

### Relationship between mortality and time in the intensive care unit

We divided the studied population into two different groups according to the median ICU LOS (5 days): long ICU stay (n = 163, 40 frail) and short ICU stay (n = 172, 37 frail). Significant differences between the two groups were noted: in patients with shorter ICU LOSs, mortality was consistently lower in the hospital and after 6 months of follow-up (Table 3).

**Table 3:** Hospital and 6-month mortality according to intensive care unit length of stay

	Hospital mortality			6-month mortality		
	Short stay (N = 172)	Long stay (N = 163)	OR (95%CI)	Short stay (N = 172)	Long stay (N = 163)	OR (95%CI)
Nonfrail (N = 173)	9 (10.3)	12 (17.4)	1.8 (0.8 - 4.4)	13 (14.9)	17 (24.4)	1.8 (0.9 - 4.0)
Prefrail (N = 85)	10 (20.8)	11 (37.8)	2.3 (0.9 - 6.1)	11 (22.9)	12 (40.5)	2.3 (0.9 - 5.8)
Frail (N = 77)	10 (27.0)	20 (50.0)	2.7 (1.0 - 7.0)	13 (35.1)	25 (62.5)	3.1 (1.2 - 7.8)
	p* = 0.052	p* = 0.042		p* < 0.001	p* < 0.001	

OR - odds ratio; 95%CI - 95% confidence interval.

* Chi-square test. Data presented as n (%).

However, this difference was more striking in frail patients (Table 3). The ORs for both hospital mortality and 6-month mortality increased from nonfrail to prefrail to frail patients.

### Variables associated with mortality

Our multivariable models revealed that the SAPS II score was associated not only with in-hospital mortality but also with 6-month all-cause mortality. The only comorbidity also associated with mortality in our models was heart failure. Curiously, both frailty and ICU LOS were associated with 6-month mortality but not with hospital mortality (Table 4).

**Table 4:** Multivariable analysis for hospital mortality and 6-month all-cause mortality

	Hospital mortality		p value
	OR	95%CI	
Prefrail*	1.41	0.69 - 2.90	0.35
Frail*	1.91	0.93 - 3.92	0.08
6-month mortality			
ICU LOS†	1.02	1.00 - 1.04	0.023
Prefrail*	1.05	0.54 - 2.07	0.883
Frail*	2.14	1.11 - 4.11	0.023

OR - odds ratio; 95%CI - 95% confidence interval; ICU - intensive care unit; LOS - length of stay.

* Control: nonfrail;

† By day.

## DISCUSSION

In this study, we aimed to analyze the relationships between frailty and ICU, hospital, and 6-month mortality. Additionally, we explored the relationship between the ICU LOS and mortality.

Frail patients were significantly older and had higher SAPS II scores, but few differences were observed in patients who needed organ support therapies. Mortality increased with frailty, either ICU, hospital, or 6-month mortality, even after adjusting for severity scores. These differences were especially pronounced in patients with long ICU stays. In addition, the need for hospital readmission in those discharged was much more common in frail patients.

An association between frailty and hospital mortality has been reported in previous studies.^(8,9)^ In our study, frailty was independently associated with 6-month all-cause mortality. Frailty represents a dimension of the patient evaluation that escapes from the severity scores traditionally used in the ICU; therefore, it could complement the SAPS II in assessing patient prognosis.^(10)^

Critical illness is associated with significant disturbances in the homeostasis of each patient, persistent inflammation, and sequelae of dysfunction after hospital discharge.^(11,12)^ Additionally, all invasive interventions during intensive care hospitalization can contribute to patient deterioration, resulting in a predictable negative long-term impact,^(13)^ which is primarily time dependent.^(14)^ Therefore, patients with lower functional reserves, including frail patients, are less tolerant of the persistence of the disease and prolonged hospitalization. This may even be more pronounced in critically ill septic patients.^(15,16)^ In addition, some patients may already present an underrecognized end-of-life trajectory that becomes apparent only during their critical illness.^(17,18)^ We have previously shown^(7)^ that frail patients were commonly treated with invasive organ support therapy and subsequently, provided a DNR order.

Accordingly, an interaction between LOS in the ICU and frailty could be expected. Our study revealed that frailty and longer durations in the ICU were independently associated with long-term mortality but not hospital mortality (Table 4). This finding attests to the potential interaction between frailty and a long ICU stay that influences the risk of a poor outcome.^(19)^ In a recently published study of patients with an ICU LOS ≥ 7 days, frailty strongly influenced 6-month mortality and quality of life.^(19)^ These findings align with our data and those of other previously published studies,^(2,6,20)^ suggesting an independent impact of frailty on outcomes. Our study stands out for its unique analysis of the relationships among frailty, ICU LOS and mortality, revealing that this association is not as evident in patients with a short ICU stay. Not only did patients with longer ICU stays have higher 6-month mortality rates than those with shorter ICU durations, but this difference between long and short ICU LOSs was more pronounced in frail patients: OR (95% CI) for frail patients 3.1 (1.2 - 7.8) *versus* for nonfrail patients 1.8 (0.9 - 4.0).

Our study has several limitations, mostly due to its relatively small sample size, which limits our conclusions. In addition, we did not address end-of-life decisions after ICU discharge or measure quality of life after hospital discharge, which may have influenced our results.

## CONCLUSION

Frailty may influence patient outcomes in the intensive care unit, including short- and long-term mortality. In our cohort, a prolonged intensive care unit length of stay was associated with a worse long-term outcome, especially in frail patients.
